# Numerical simulation of foundation pit dewatering using horizontal seepage reducing body

**DOI:** 10.1038/s41598-022-05348-y

**Published:** 2022-01-26

**Authors:** Jianxiu Wang, Yanxia Long, Yu Zhao, Xiaotian Liu, Weiqiang Pan, Jianxun Qu, Hanmei Wang, Yujin Shi

**Affiliations:** 1 Department of Geotechnical Engineering, College of Civil Engineering, Tongji University, Shanghai, 200092 China; 2 Key Laboratory of Geotechnical and Underground Engineering of Ministry of Education, Tongji University, Shanghai, China; 3 Shanghai Tunnel Engineering Company Co., Ltd., Shanghai, 200082 China; 4 Shanghai Institute of Geological Survey, Shanghai, 200093 China

**Keywords:** Hydrology, Engineering

## Abstract

Groundwater level has to be lowered during deep excavation. A vertical curtain is usually adopted to control the drawdown inside and outside a foundation pit in a built-up area. However, the cost and working difficulty increases substantially with the rise in depth of vertical curtains. In the manuscript, a man-made horizontal seepage reducing body (HSRB) was introduced to shorten the vertical curtain depth and control drawdown. With the No. 4 shaft foundation pit of Guangyuan Project, Shanghai as background, HSRB was proposed in foundation pit dewatering. Microbially induced carbonate precipitation grouting technology was recommended to form an environment-friendly HSRB. Numerical method was used to simulate and understand the influence of position, thickness, and hydraulic conductivity of HSRB on groundwater level. The non-separated HSRB was better than the separate HSRB. Decreasing HSRB hydraulic conductivity was better than increasing HSRB depth. Four seepage modes were summarized considering vertical curtain penetration conditions into multi-aquifer, and the fifth seepage mode was formed for vertical curtain using man-made HSRB, which can be referred by similar engineering applications.

## Introduction

Coastal cities are often developed in the economy because of convenient transportation. Urbanization develops quickly. Underground space is developed to serve the development of the city. In the aspect of engineering geology, engineering hydrogeology is important for a coastal city. Multi-aquifer and multi aquitard are often encountered during underground exploitation. Dealing with the multi-aquifer is important for a deep excavation. Meanwhile, controlling the environment influence of lowering ground water level is also important. The excavation depth is continuously increased under the urbanization demand, and the required drawdown is increasing correspondingly. The contradiction between the increasing drawdown and strict requirements of surrounding land subsidence, as well as groundwater resource protection, is also expanding. The majority of the accidents in foundation pit are concerned with groundwater. Controlling groundwater level effectively during excavation has become a major research task^1–7^. The improper control of groundwater in excavation and construction processes often leads to large ground deformation^8–10^, damage to surrounding buildings^11,12^, quicksand and gushing^13–15^, and other engineering hazards. Field monitoring has indicated that the pumping and depressurization of confined water are the main factors causing ground settlement in foundation pit engineering^16,17^. The influence range reaches 10 to 15 times of the excavation depth^18^. Therefore, groundwater control in excavation is necessary to ensure the safety of foundation pit and environment.

**Figure 1:**
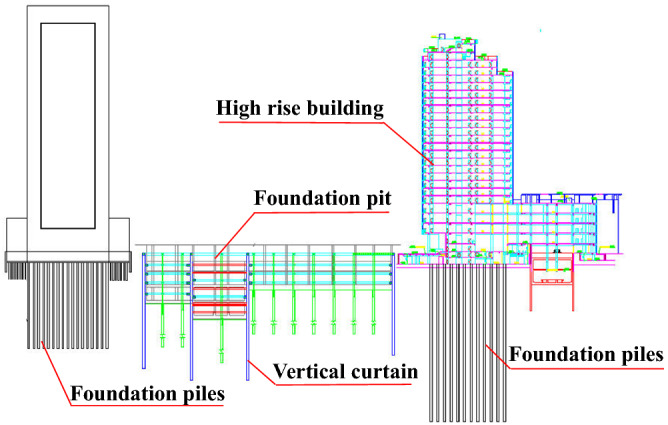
Curtain cutoff, pumping and recharging measures to control groundwater level for excavation.

At present, curtain cutoff, pumping, and recharging are widely used in groundwater control of a foundation pit (Fig. 1). Pumping is generally used and curtain is currently utilized to achieve foundation pit dewatering^19–21^. Vertical curtain is usually used to cut off aquifers, decrease aquifer discharge section, change seepage direction, and prolong seepage path. Some studies have evaluated the dewatering effect of the insertion depth of vertical curtain^15,22–25^, pumping rate^26^, hydraulic conductivity, and distance between pumping well and vertical curtain^20,21^. Wang et al.^2^ analyzed the mode and mechanism of the wall–well interaction. As a result, four patterns including fully enclosed, flush, partially enclosed, and fully exposed types are defined. The depth of vertical curtain penetrating into aquifer influence drawdown obviously. The interaction between vertical curtain and pumping well should be considered in foundation pit dewatering (i.e., wall–well interaction)^15^. The application of the wall–well interaction in different projects was also summarized^2,27–30^. However, the verticality of curtain, such as diaphragm wall, is difficult to be controlled precisely when the depth of vertical curtain is too large. If the verticality is not controlled in a certain value, then the foot of two adjacent diaphragm wall splits leading to water leakage, which is dangerous for groundwater control. The vertical curtain cannot cut off aquifers and cannot meet the strict settlement control requirements of the surrounding environment when the aquifer is too deep. This type of deep confined aquifers has led to the use of horizontal curtain^31^. However, horizontal curtain cannot avoid local leakage owing to complex hydrogeological conditions and construction quality. Local leakage points result in water inrush.

In the manuscript, a man-made horizontal aquiclude with lower permeability was introduced in the dewatering system with vertical curtain. A man-made Horizontal Seepage Reducing Body (HSRB) was proposed. With the No. 4 shaft foundation pit of the Guangyuan Project, Shanghai as background, HSRB was suggested to combine with vertical curtain to control groundwater drawdown. Microbially Induced Carbonate Precipitation (MICP) grouting technology was suggested to form the HSRB. Finite Difference Method (FDM) was used to simulate the working mechanism of HSRB. The position, thickness, and hydraulic conductivity of HSRB were analyzed, which can be referred by similar projects.

## Material and methods

### Project overview

**Figure 2:**
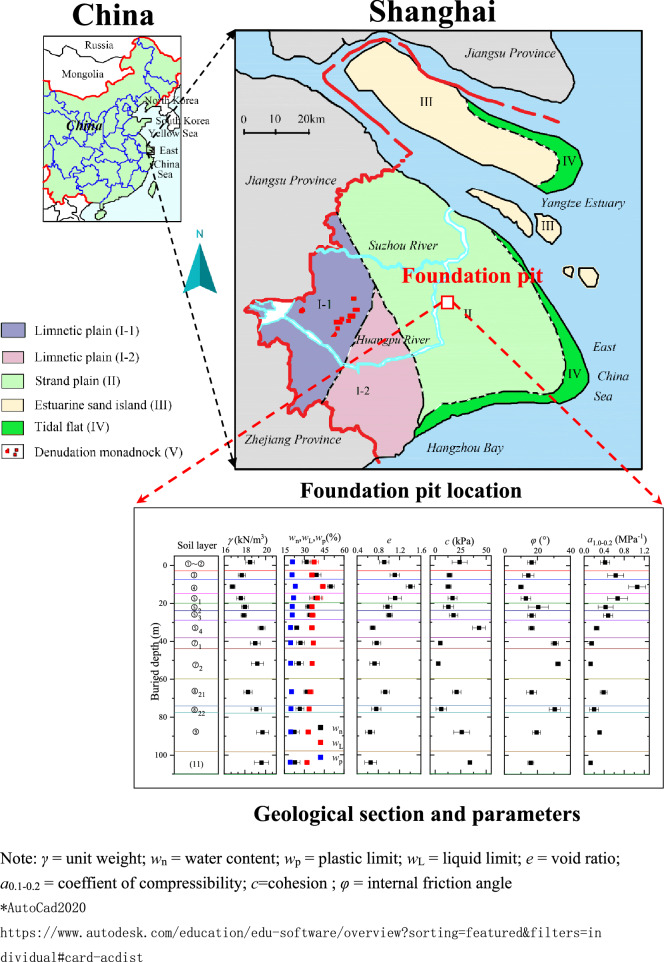
Layout of the No. 4 working shaft background.

The No. 4 shaft foundation pit (Fig. 2) of Guangyuan Project in Pudong New District, Shanghai is located on Jihui Road of Gaoyan Institute, 97.1 m away from the West 220 kV high-voltage iron tower, 12 m away from the east substation, and 8.5 m nearest to a two-story pump house. The surrounding environment of the shaft was complicated. The foundation pit is a 55 m × 50 m rectangular in plane. The designed ground elevation is 4.5 m, while the pit excavation depth is 39.6 m. The 150 m depth layers of the site was composed of the Quaternary Holocene to Middle Pleistocene sedimentary strata. The strata are divided into 13 main engineering geological layers (Fig. 2b). From top to bottom, the strata are divided into layer ①‒②, layer ③, layer ④, layer ⑤_1_, layer ⑤_2_ (clayey silt with silty clay), layer ⑤_3_ (silty clay) , layer ⑤_4_ (silty clay ), layer ⑦_1_ (sandy silt ), layer ⑦_2_ (silt), layer ⑧_21_ (interlayer of silty clay and silt), layer ⑧_22_ (silty sand with silty clay), layer ⑨ (silt) and layer (11)(silt). The layer ⑤_1_ and above layers were generalized as shallow soil layers. The foundation pit bottom was located in the silty clay of layer ⑤. The enclosure retaining system composed of diaphragm wall, outer Trench cutting Re-mixing Deep wall (TRD), and inner support. The diaphragm wall was 1200 mm thick and 86 m deep, and the toe of the wall entered the layer ⑨ approximately 6 m. Diaphragm wall was also used as vertical curtain for dewatering.

The aquifers consisted of phreatic aquifer (shallow soil layers), micro confined aquifer (layer ⑤,) confined aquifer I (layer ⑦), confined aquifer II (layer ⑨), and confined aquifer III (layer ⑾). The recommended hydraulic conductivities for each layer based on laboratory and in-site pumping test are shown in Table 1.

**Table 1:** Hydraulic parameter of the soil layers.

Layer	Soil	Hydraulic conductivity (cm/s)			Storage coefficient S_s_ (1/m)
		Vertical K_v_	Horizontal K_h_ (cm/s)	Recommended isotropic K (cm/s)	
/	Shallow clay layer	2.21 × 10^–7^	3.69 × 10^–7^	5.0 × 10^–5^	8.0 × 10^–4^
⑤_2_	Clayey silt with silty clay	1.25 × 10^–4^	1.97 × 10^–4^	3.0 × 10^–4^	4.5 × 10^–4^
⑤_3_	Silty clay	7.07 × 10^–5^	8.97 × 10^–5^	8.0 × 10^–5^	8.0 × 10^–3^
⑤_4_	Silty clay	3.46 × 10^–7^	4.17 × 10^–7^	4.0 × 10^–5^	8.0 × 10^–3^
⑦_1_	Sandy silt	1.04 × 10^–7^	1.70 × 10^–7^	1.48 × 10^–3^	4.5 × 10^–4^
⑦_2_	Silt	4.21 × 10^–4^	5.84 × 10^–4^	1.40 × 10^–3^	5.0 × 10^–4^
⑧_21_	Interlayer of silty clay and Silt	4.60 × 10^–5^	5.80 × 10^–4^	6.0 × 10^–5^	4.5 × 10^–4^
⑧_22_	Silty sand with silty clay	1.00 × 10^–3^	6.40 × 10^–3^	6.4 × 10^–3^	5.0 × 10^–4^
⑨	Silt	1.00 × 10^–2^	9.00 × 10^–2^	5.0 × 10^–2^	2.0 × 10^–5^
⑾	Silt	5.00 × 10^–3^	4.90 × 10^–2^	1.0 × 10^–2^	3.0 × 10^–5^

The foundation pit bottom was mainly located in clayey soil. Dewatering schemes for each aquifer under the pit were arranged as shown in Table 2.

**Table 2:** Statistical table for the layout of dewatering wells in foundation pit.

Position	Well type	Soil layers	Depth	Quantity	Aperture/well diameter/well pipe thickness	Well No	Thickness of clay ball (m)
In pit	Dewatering well	① ~ ⑤_3_	38	12	650/273/4 mm	4 J-1 to 4 J-12	0
	Depressurization well	⑦ ~ ⑧_21_	68	1	650/273/6 mm	4Y7-1	8
	Depressurization well		63	1	650/273/6 mm	4Y7-2	8
	Observation well		60	1	650/273/6 mm	4G7-1	8
	Observation well		70	1	650/273/6 mm	4G8-1	8
	Depressurization well	⑧_22_ ~ ⑨	85	3	850/400/8 mm	4Y9-1 to 4Y9-3	10
	Spare well		85	1	850/400/8 mm	4YB9-1	
	Observation well		85	1	850/400/8 mm	4G9-1	
Between two walls	Spare and observation well	⑤_2_	30	4	650/273/4 mm	4JG52-1 to 4JG52-4	0
	Spare and observation well	⑦	63	4	650/273/6 mm	4JG7-1 to 4JG7-4	8
Out pit	Observation well	⑦	60	4	650/273/6 mm	4WG7-1 to 4WG7-4	8
	Observation well	⑧_21_	70	2	650/273/6 mm	4WG8-1 to 4WG8-2	8
	Observation well	⑧_22_ ~ ⑨	90	1	850/325/6 mm	4WG9-1	8

### Definition of HSRB

HSRB is a kind of horizontal seepage reducing body with a certain thickness formed by reducing the hydraulic conductivity of soil by grouting in confined aquifer. It can reduce seepage in foundation pit dewatering, increase hydraulic gradient in curtain body, reduce outlet pressure and effectively control seepage. This method is economical, simple and easy to realize compared with the complete impermeable curtain.

An MICP man-made HSRB was suggested besides the vertical curtain consisted by diaphragm wall and TRD to reduce the influence on surrounding environment. MICP is a biologically induced mineralization of calcite type calcium carbonate crystals with excellent cementation by providing specific microorganisms with abundant Ca^2+^ sources and nitrogen-containing nutrients. MICP grouting technology was suggested to form the HSRB using *Bacillus pasteurella* and cementing fluid (CaCl_2_ solution, urea solution).

### Numerical modeling

The hydrogeological conceptual model was translated into a mathematical model. The hydraulic parameters were inversed and verified on the basis of pumping tests by using numerical method during hydrological investigation^32,33^. A three-dimensional (3D) unsteady flow continuity equation was established in anisotropic porous media:

1 $$\left\{ {\begin{array}{*{20}l} {\frac{\partial }{\partial x}\left( {k_{xx} \frac{\partial h}{{\partial x}}} \right) + \frac{\partial }{\partial y}\left( {k_{yy} \frac{\partial h}{{\partial y}}} \right) + \frac{\partial }{\partial z}\left( {k_{zz} \frac{\partial h}{{\partial z}}} \right) - W = \frac{E}{T}\frac{\partial h}{{\partial t}}} \hfill & {(x,y,z) \in \Omega } \hfill \\ {h\left( {x,y,z,t} \right)\left| {_{t = 0} = h_{0} \left( {x,y,z,{\text{t}}} \right)} \right.} \hfill & {(x,y,z) \in \Omega } \hfill \\ {h\left( {x,y,z,t} \right)\left| {_{{\Gamma_{1} }} = h_{1} \left( {x,y,z,t} \right)} \right.} \hfill & {(x,y,z) \in \Gamma_{1} } \hfill \\ {q\left( {x,y,z,t} \right)\left| {_{{\Gamma_{2} }} = q_{1} \left( {x,y,z,t} \right)} \right.} \hfill & {(x,y,z) \in \Gamma_{2} } \hfill \\ \end{array} } \right.$$

where $$E = \left\{ \begin{gathered} S\;\;\;\;{\text{Confined aquifer }} \hfill \\ S_{y} \;\;\;{\text{Diving aquifer }} \hfill \\ \end{gathered} \right.$$; $$T = \left\{ \begin{gathered} M\;\;\;\;{\text{Confined aquifer }} \hfill \\ B\;\;\;\;\;{\text{Diving aquifer }} \hfill \\ \end{gathered} \right.$$; $S_{s} = \frac{S}{M}$; $S$ is water storage coefficient, $S_{y}$ is water supply, $S_{s}$ is water storage rate $\left( {\text{1/m}} \right)$; $M$ is unit thickness of the confined aquifer $\left( {\text{m}} \right)$; $B$ is saturated thickness of groundwater in the phreatic aquifer unit body $\left( {\text{m}} \right)$; $k_{xx} ,k_{yy} ,k_{zz}$ are the anisotropic principal direction hydraulic conductivities $\left( {\text{m/d}} \right)$; $h$ is head value of point $\left( {x,y,z} \right)$ at time t $\left( {\text{m}} \right)$; $W$ is source and exchange items $\left( {\text{1/d}} \right)$; $h_{0}$ is initial head value of the calculation domain $\left( {\text{m}} \right)$; $h_{1}$ is value of the water head around the Dirichlet boundary $\left( {\text{m}} \right)$; ${\text{q}}_{1}$ is flux value of the Neumann boundary of the foundation pit $\left( {\text{m}} \right)$; $t$ is time $\left( {\text{d}} \right)$; and $\Omega$ is computational domain; ${\Gamma }_{1}$ is Dirichlet boundary; ${\Gamma }_{2}$ is Neumann boundary.

**Figure 3:**
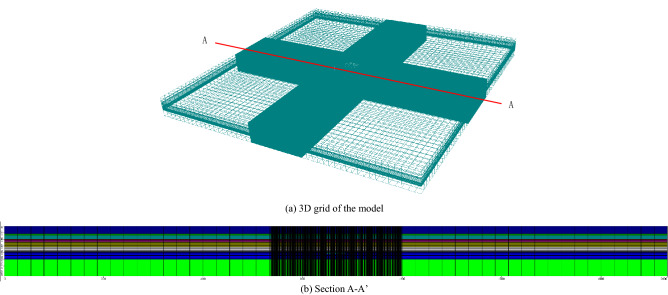
Numerical model of the MICP HSRB.

With the shaft foundation pit as center, a 2000 m × 2000 m and 150 m deep modeling range was selected. The range was generalized into a 3D heterogeneous, horizontally isotropic, and unstable groundwater seepage system. As shown in Fig. 3, the model was divided into 10 layers according to soil layer distribution. Grid in the model divided the plane into 50 × 50 grids, and then each row and column of the grid within 3 times the width of the foundation pit were further refined into 40 copies. The ground elevation was taken as + 4.5 m. The outer boundary was defined as constant water head boundary, and the bottom was set as impermeable boundary. Model hierarchy and its parameters are shown in Table 1.

For the shaft foundation pit was too deep, the confined aquifer II which was seldom concerned previously had to be lowered. Although double vertical curtains were adopted, the layer ⑨ was not cut off. MICP HSRB was suggested to control the drawdown, and it was discussed in another manuscript^*^^34^. According to laboratory results, the hydraulic conductivity of the layer ⑨ was decreased from 2.1 × 10^−3^ cm/s to 1.9 × 10^−4^ cm/s using the MICP technology.

In working conditions, the influence of the position, thickness, and permeability of the horizontal curtain on the foundation pit dewatering was designed. The working conditions of numerical simulation are shown in Table 3.

**Table 3:** Working conditions of the influence of 3D curtain on deep foundation pit dewatering.

Working condition	Depth of vertical curtain (m)	Buried depth of HSRB roof (m)	Thickness of HSRB (m)	Hydraulic conductivity of HSRB (cm/s)
1	86	/	/	/
2		82	4	5 × 10^–3^
3		84	4	5 × 10^–3^
4		86	4	5 × 10^–3^
5		88	4	5 × 10^–3^
6		90	4	5 × 10^–3^
7		92	4	5 × 10^–3^
8		82	4	5 × 10^–3^
9		82	4	5 × 10^–3^
10		82	3	5 × 10^–3^
11		82	5	5 × 10^–3^
12		82	6	5 × 10^–3^
13		82	6	1 × 10^–2^
14		82	6	1 × 10^–4^
15		82	6	5 × 10^–4^

FDM was used to solve the problem. MODFLOW^35^ was solved by FDM, and the conjugate gradient method (PCG) was chosen to simultaneously solve the algebraic equations. Groundwater level changes inside and outside the foundation pit were simulated through the calculation of MODFLOW software.

## Results

### Results without HSRB

**Figure 4:**
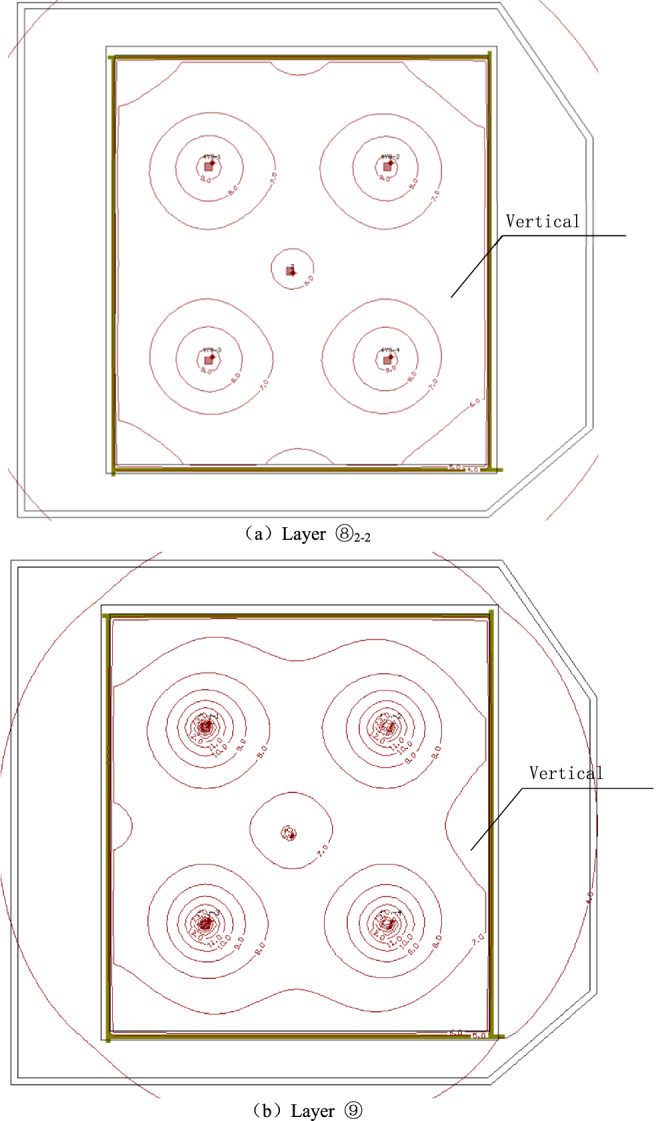
Contour map of the aquifer drawdown.

**Figure 5:**
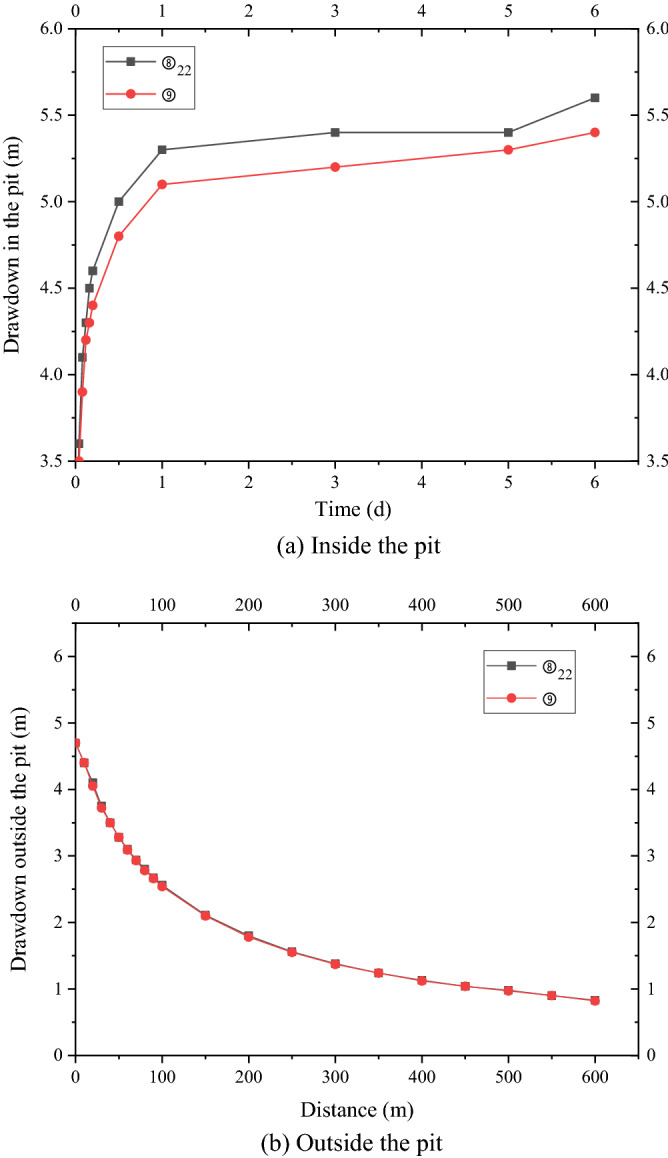
Variation curve of the drawdown with distance.

In case 1, the four wells were used to pump water simultaneously, and the pumping rate of each well was 2950 m^3^/d. After continuous pumping for 6 days, the dynamic water level of the fourth pumping wells in layer ⑧_22_ of the pit decreased by 9.0 m, and the minimum drawdown in the foundation pit was 5.56 m, which met the requirements of the foundation pit anti-gushing calculation (Fig. 4a). The requirement of drawdown of layer ⑧_22_ against inrush was 5.5 m. The change in the minimum water level with the decrease in time in the pit is shown in Fig. 5. At this time, water level in the range of 600 m of layer ⑧_22_ outside the pit decreased by 4.70 m to 0.83 m. When the dewatering of layer ⑧_22_ met the drawdown requirement (i.e., continuous pumping for 6 days), the drawdown of four wells in layer ⑨ in the pit was 12.0 m, and the minimum water level drawdown of layer ⑨ in the pit was 5.3 m, which met the requirement of 2.3 m drawdown of layer ⑨ in the foundation pit anti-gushing calculation (Fig. 4b). The variation in the minimum water level drawdown with time in the pit is shown in Fig. 5a. At this time, the water level within 600 m of layer ⑨ outside the pit decreased by 4.70 m to 0.82 m, as shown in Fig. 5b.

As shown in Fig. 5a, the water level of the deep foundation pit of the No. 4 working well decreased rapidly in the first day, which reached 5.3 m, under the simultaneous action of four pumping wells. However, the design drawdown meeting the anti-gushing calculation can be achieved on the sixth day owing to the large permeability of the second confined aquifer in Shanghai and the large pumping rate of the foundation pit. As shown in Fig. 5b, the drawdown of the water level outside the foundation pit of layer ⑧_22_ and layer ⑨ coincided with the distance, and the change in the drawdown curve can be divided into three areas. (1) Within the range of 0–150 m, the drawdown gradient with the distance was large, and the change in water level was large. (2) Within the range of 150–300 m, the gradient of the drawdown with the distance began to decrease, and the change in water level was slow and gradually transited to the slow area. Beyond the range of 300 m, the drawdown gradient with distance was small, and the change in water level was small as well.

### Influence of HSRB position on dewatering

The depth of the HSRB setting should be determined before forming a HSRB by using the MICP technology. The HSRB is normally suggested to be installed in the balance position between the weight of overlying soil layers and underlying water pressure of confined aquifers after the excavation of the foundation pit. The calculation formula was as follows:

2 $$\sum {\gamma_{i} h_{i} \ge \gamma_{s} } \gamma_{w} H,$$

where $\gamma_{i}$ is weight of each soil layer (kN/m^3^); $h_{i}$ is thickness of each layer (m); $H$ is pressure head height at the horizontal curtain (m); $\gamma_{w}$ is water severity (kN/m^3^), which has a value of 10 kN/m^3^; and $\gamma_{s}$ is safety coefficient, which has a value of 1.05.

The weight and the thickness of each layer are given in Fig. 2b. By calculating Formula (2), the value of *H* obtained as 82 m. Thus, the buried depth of the horizontal curtain was 82 m.

The influence of HSRB at different positions on deep foundation pit dewatering was studied in accordance with the calculation results of working conditions 2 to 7, as well as the comparative analysis of the calculation results of condition 1. The thickness of HSRB was 4 m, and the hydraulic conductivity was 5 × 10^−3^ cm/s. According to the different positions of the horizontal curtain, the combination forms of the 3D curtain were classified into inner-wrapping, flush, and separated types, as shown in Table 4.

**Table 4:** Combination forms of three dimensional curtain.

Working condition	Buried depth of HSRB roof (m)	Thickness of HSRB (m)	Hydraulic conductivity (cm/s)	The form of three- dimensional curtain
1	/	4	5 × 10^–3^	/
2	82	4	5 × 10^–3^	Inner-wrapped type
3	84	4	5 × 10^–3^	Transitional type
4	86	4	5 × 10^–3^	Flush type
5	88	4	5 × 10^–3^	Separated type
6	90	4	5 × 10^–3^	Separated type
7	92	4	5 × 10^–3^	Separated type

**Figure 6:**
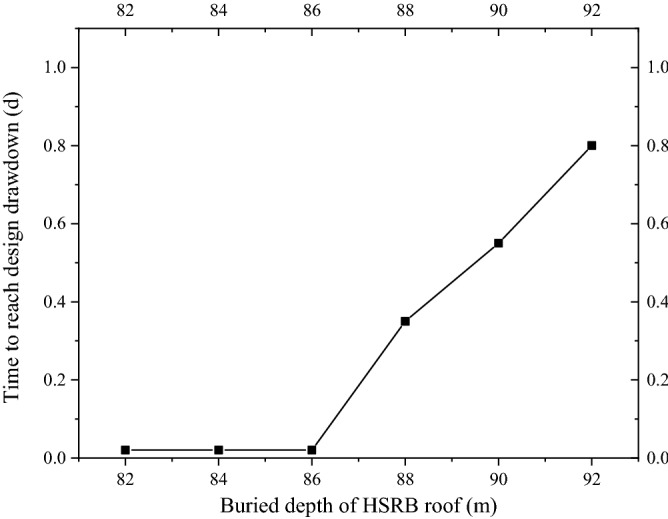
Time chart of the dewatering reaching the design depth of each working condition.

Four pumping wells (i.e., 4y9-1 to 4y9-4) were used to pump water simultaneously in conditions 2 to 7, and the pumping rate of the four wells was 2950 m^3^/d. The time required for the drawdown of the water level in the pit to reach the design drawdown can be obtained, as shown in Fig. 6, from the calculation results. When the buried depths of HSRB were 82, 84, and 86 m, the HSRB was not separated from the vertical curtain. In these cases, the time required for the water level in the pit to reach the design water level of anti-inrush calculation of the foundation pit was substantially shortened compared with that without HSRB. It was approximately 0.4% of that without HSRB. For several working conditions of the non-separated 3D curtain, the position and depth of horizontal curtain had minimal influence on the dewatering time of the deep foundation pit.

When the buried depths of the horizontal curtain were 88, 90, and 92 m (i.e., HSRB and vertical curtain were separated and combined to form separate 3D curtain), the times required to reach the design drawdown were 0.35, 0.55, and 0.8 d, respectively. Compared with the non-separated 3D curtain, the time to reach the design drawdown was increased. However, the time to reach the design drawdown was also significantly shorter than that without HSRB, which was only approximately 10% of that without HSRB. With the deepening of the buried depth of the horizontal curtain, the time required for the foundation pit dewatering to reach the design drawdown increased correspondingly.

Therefore, from the perspective of dewatering time, the effect of setting HSRB on the design drawdown of the foundation pit dewatering was significant, and the effect of the non-separated 3D curtain was better than that of the separation three-dimensional curtain. The work efficiency of setting horizontal curtain was evidently higher than that without HSRB. In practical engineering, the construction period was substantially reduced, construction efficiency was considerably improved, construction nodes can be completed in advance, and good social and economic benefits will be achieved.

**Figure 7:**
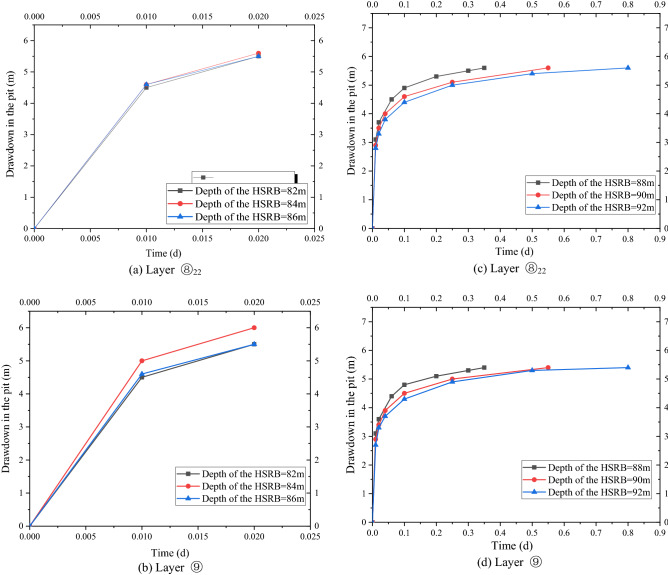
Drawdown–time curve in the pit with different HSRB positions.

The non-separated and separated 3D curtain were studied separately to significantly compare the influence of the different types of 3D curtain on deep foundation pit dewatering. Working conditions 2 to 4 were calculated to study the influence of the position of the non-separating horizontal curtain on the 3D curtain–well group system. The minimum drawdown of the water levels in the pit of layers ⑧_22_ and ⑨ are shown in Fig. 7a,b, respectively. The drawdown time curve under the working conditions of 82, 84, and 86 m of the horizontal curtain had minimal difference. After 28.8 min of pumping well operation, the drawdown of the water level in the pit reached 5.6 m of the design requirement.

Working conditions 4 to 6 were calculated to study the influence of the position of the separated horizontal curtain on the 3D curtain–well group system. The minimum drawdown of the water levels in the pit of layers ⑧_22_ and ⑨ are shown in Fig. 7c,d, respectively. The time required to reach the design drawdown in the pit increased with the deepening of HSRB. Before the design drawdown was reached, the minimum drawdown in the foundation pit under the corresponding working conditions was smaller when the HSRB was buried deeper.

During the excavation of the deep foundation pit in Shanghai, the main purpose of extracting groundwater from the deep second confined aquifer was to reduce the water head pressure at the bottom of the pit and avoid the occurrence of sudden gushing at the bottom of the pit. However, the decrease in the groundwater level leads to an increase in the effective self-weight stress of the layer below the original water level, soil consolidation, ground settlement around the foundation pit, and uneven settlement, inclination, and cracking of underground pipelines and surface buildings. Therefore, effective measures should be implemented to reduce or even eliminate the impact of foundation pit dewatering on the surrounding environment. The setting of vertical curtain relatively reduced the impact of foundation pit dewatering on the surrounding environment. However, merely setting a vertical curtain may not meet the requirements for engineering with strict requirements on the surrounding environment. Thus, HSRB should be added to further eliminate the settlement of the pit bottom caused by foundation pit dewatering. Therefore, the influence of HSRB on groundwater level outside the pit must also be referred to evaluate the effect of the different HSRB position, thickness, and hydraulic conductivity on the 3D curtain–well group system on the deep foundation pit dewatering engineering.

**Figure 8:**
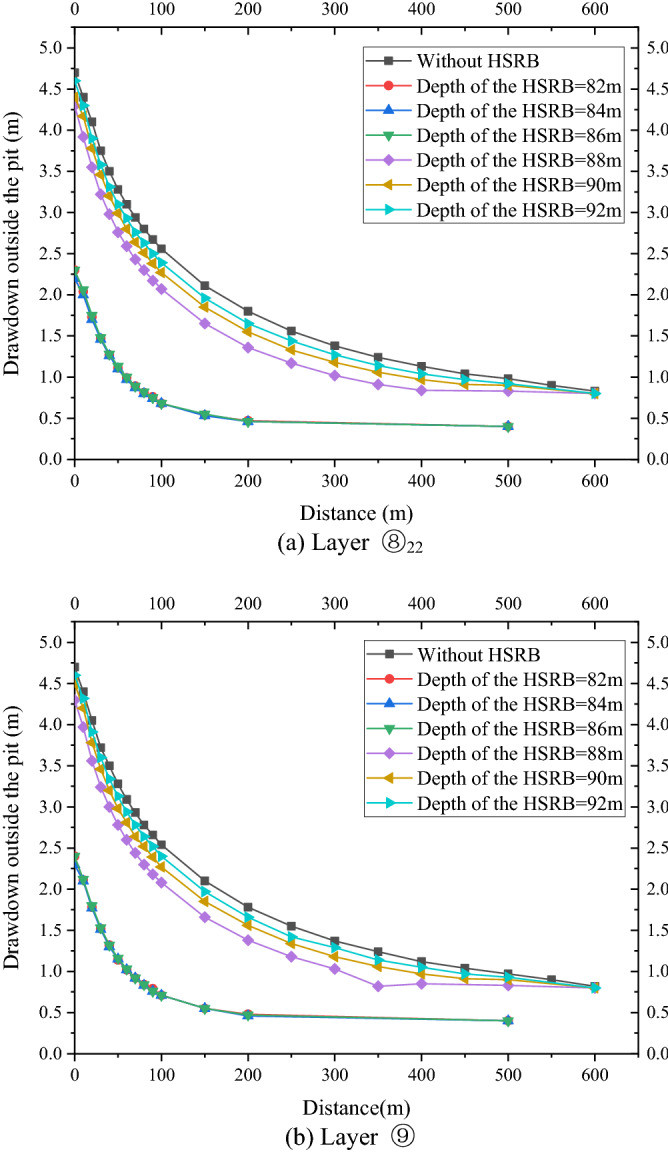
Drawdown–distance curve outside the pit with different HSRB positions.

As shown in Fig. 8a,b, the variation law of the drawdown distance curve outside the pit of layers ⑧_22_ and⑨ under each working condition was consistent. Meanwhile, the drawdown value and drawdown trend of the water levels outside the pit of layers ⑧_22_ and ⑨ were the same. Therefore, this study only analyzed the drawdown variation outside the pit of layer ⑧_22_ with distance. For the working condition of non-separated 3D curtain, the drawdown distance curves of the three working conditions were nearly coincidental when the HSRB buried depths were 82, 84, and 86 m. When the drawdown of water level in the pit reached the design requirement of 5.52 m, the maximum drawdown of the water level outside the pit was 2.4 m, and the drawdown of the water level outside the pit was 0.5 m when the drawdown of the water level outside the pit was 150 m. For the non-separated 3D curtain, the position of the HSRB in the 3D curtain had evident minimal influence on the deep foundation pit dewatering project, which can be disregarded.

For the working conditions of the separated curtain (i.e. when HSRB buried depths were 88, 90, and 92 m), the variation trend of the drawdown distance curve outside the pit under the three working conditions was consistent, and the curve slope was the same. That is, the drawdown rate of the water level outside the pit was the same with a decrease in distance. However, the drawdown of the water level outside the pit was different with the varying positions of HSRB. The drawdown of the water level outside the pit was higher when the HSRB was buried deeper, and the impact on the environment was greater when the settlement of the ground outside the pit was greater. The maximum drawdown of the water level outside the pit was 4.3 m and the maximum drawdown 400 m away from the pit was 0.84 m when HSRB depth was 88 m. The maximum drawdown of the water level outside the pit was 4.5 m and the maximum drawdown 450 m away from the pit was 0.91 m when HSRB depth was 90 m. The maximum drawdown of the water level outside the pit was 4.6 m and the maximum drawdown 500 m away from the pit was 0.92 m when the HSRB depth was 92 m.

In summary, for the non-separated type of 3D curtain, the effects of the inner- wrapped, flush, and transitional 3D curtains on the deep foundation pit dewatering engineering were equivalent, and they were better than the effect of the separated type 3D curtain. In the separated 3D curtain, the effect was better when the horizontal curtain is closer to the bottom of the vertical curtain. Therefore, the design form of non-separated 3D curtain should be adopted in practical engineering.

### Influence of HSRB thickness on dewatering

From the analysis results of the influence of HSRB position on the 3D curtain–well group system, the effect of the non-separated HSRB was better than that of the separated horizontal curtain, and the influence of the HSRB position on the dewatering effect of the internal 3D curtain was minimally evident. The MICP technology was used to form a horizontal curtain, which involved the seepage of bacteria and cementation liquids in groundwater, to avoid the impact of bacteria and cementation liquids used in MICP on the surrounding environment. Thus, vertical curtain was used to control the MICP bacteria and cementation liquids within the scope of the vertical curtain of foundation pit. Therefore, the thickness of the MICP bacterial liquid infusion was not over 6 m when the buried depth of the horizontal curtain roof was set at 82 m. Thus the impact on the surrounding environment was decreased.

If the pumping rate of 2950 m^3^/d was the same as that of conditions 2 to 7, and the horizontal curtain thickness was increased, then dewatering in the foundation pit rapidly reached the designed drawdown. Difficulty is encountered in analyzing the relationship between the drawdown of HSRB with different thicknesses and times. Therefore, the pumping rate of the pumping well should be reasonably reduced. Designing conditions 8, 9, and 2 were compared and analyzed to evaluate the influence of pumping rate of the pumping well on the dewatering of the deep foundation pit. Only the pumping rate was different under the three conditions, and other conditions were the same. The specific parameters are shown in Table 5.

**Table 5:** Working conditions of different pumping rates.

Working condition	Buried depth of HSRB roof (m)	Thickness of HSRB (m)	Hydraulic conductivity of HSRB (cm/s)	Pumping rates (m^3^/d)
2	82	4	5 × 10^–3^	2950
8	82	4	5 × 10^–3^	2500
9	82	4	5 × 10^–3^	2000

**Figure 9:**
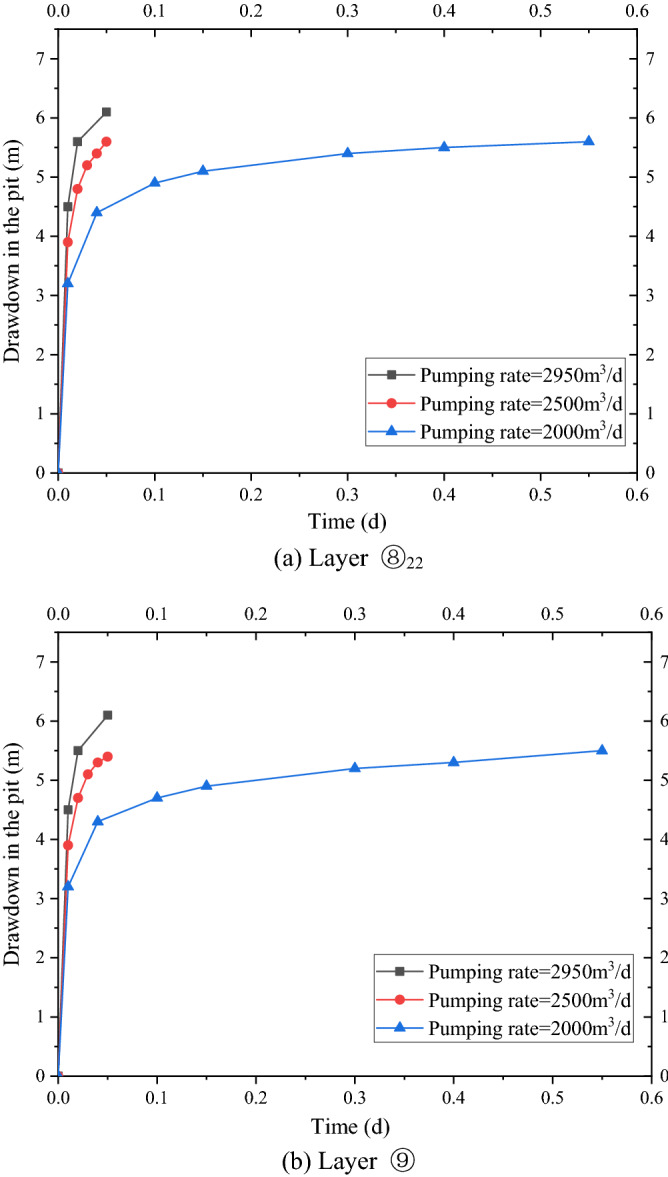
Drawdown–time curve in the pit with different pumping rates.

**Figure 10:**
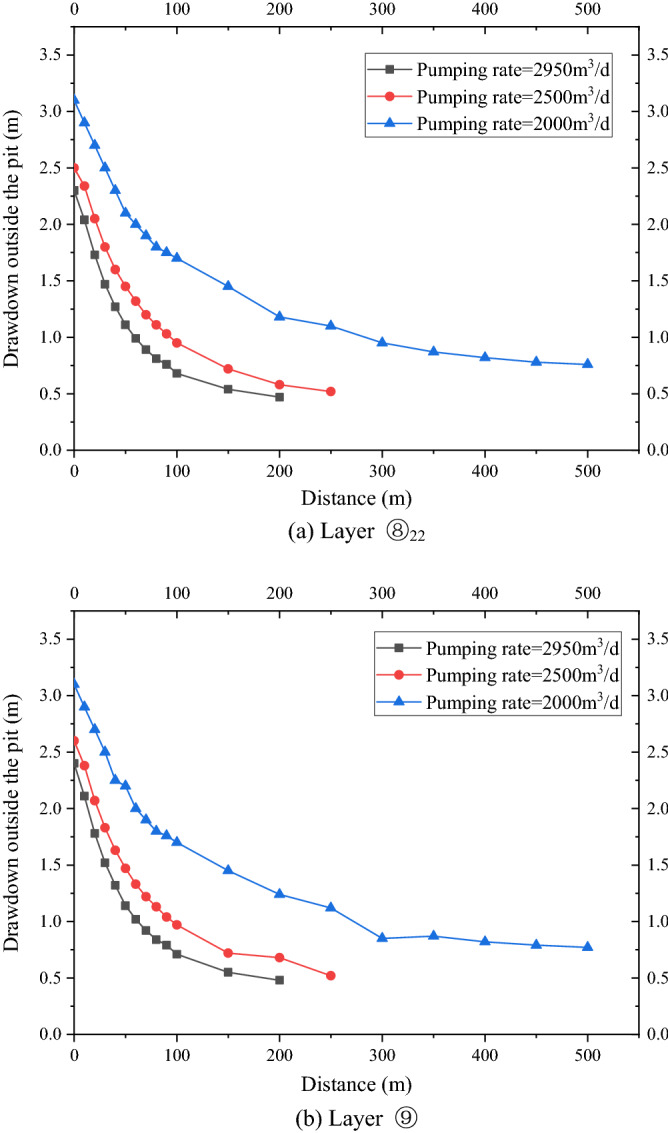
Drawdown–distance curve outside the pit with different pumping rates.

The drawdown-to-time curves of the water level in the pit under three conditions of the different pumping rates based on the numerical simulation results are shown in Fig. 9a,b, and the drawdown-to-distance curves outside the pit are shown in Fig. 10a,b. As shown in Fig. 9a,b, the time required for the water level in the pit to reach the design drawdown increased when the pumping rate of the pumping well decreased from 2950 m^3^/d to 2500 m^3^/d. A total of 28.8 min was needed to reach the design drawdown when pumping rate was 2950 m^3^/d. A total of 72 min was needed to reach the design drawdown when pumping rate was 2500 m^3^/d. A total of 72 min was needed to reach the design drawdown when pumping rate was 2000 m^3^/d.

As shown in Fig. 10a,b, the drawdown value and trend of the water level outside the pit of the two layers were the same. Therefore, only the variation in the drawdown of the water level outside the pit of the two layers with the distance was analyzed. The maximum drawdown was 2.3 m when pumping rate was 2950 m^3^/d, and it reached a stable drawdown of 0.48 m at 200 m away from the pit. The maximum drawdown was 2.5 m when pumping rate was 2500 m^3^/d, and it reached a stable drawdown of 0.52 m at 200 m away from the pit. The maximum drawdown was 3.1 m when pumping rate was 2000 m^3^/d, and it reached a stable drawdown of 0.75 m at 300 m away from the pit. The maximum drawdown of the water level outside the pit increased and the distance to reach the stable drawdown level also increased with a decrease in pumping rate. That is, the impact on the surrounding environment increased substantially.

In summary, different pumping rates influenced the effect of deep foundation pit dewatering. The time to reach the designed drawdown level was longer and the larger the drawdown and influence range of the water level outside the pit was larger when the pumping rate was lower. Therefore, a high pumping rate is beneficial to the deep foundation pit dewatering project, although a necessary action is to set a reasonable pumping rate that considers the actual situation of the construction site.

The calculation of working conditions 8, 10, 11, and 12 was performed to study the influence of HSRB thickness on the 3D curtain–well group system. Four pumping wells in each working condition pump water at a pumping rate of 2500 m^3^/d. The buried depth of the horizontal curtain roof was 82 m, and the horizontal curtain thicknesses in each working condition were 3, 4, 5, and 6 m. The 3D curtain formed by the horizontal and vertical curtains was inner-wrapped 3D curtain, and the hydraulic conductivity was 5 × 10^−3^ cm/s. The specific parameters are shown in Table 6.

**Table 6:** Working conditions of different HSRB thicknesses.

Working condition	Buried depth of HSRB roof (m)	Thickness of HSRB (m)	Hydraulic conductivity of HSRB (cm/s)	Pumping rates (m^3^/d)
8	82	4	5 × 10^–3^	2500
10	82	3	5 × 10^–3^	2500
11	82	5	5 × 10^–3^	2500
12	82	6	5 × 10^–3^	2500

**Figure 11:**
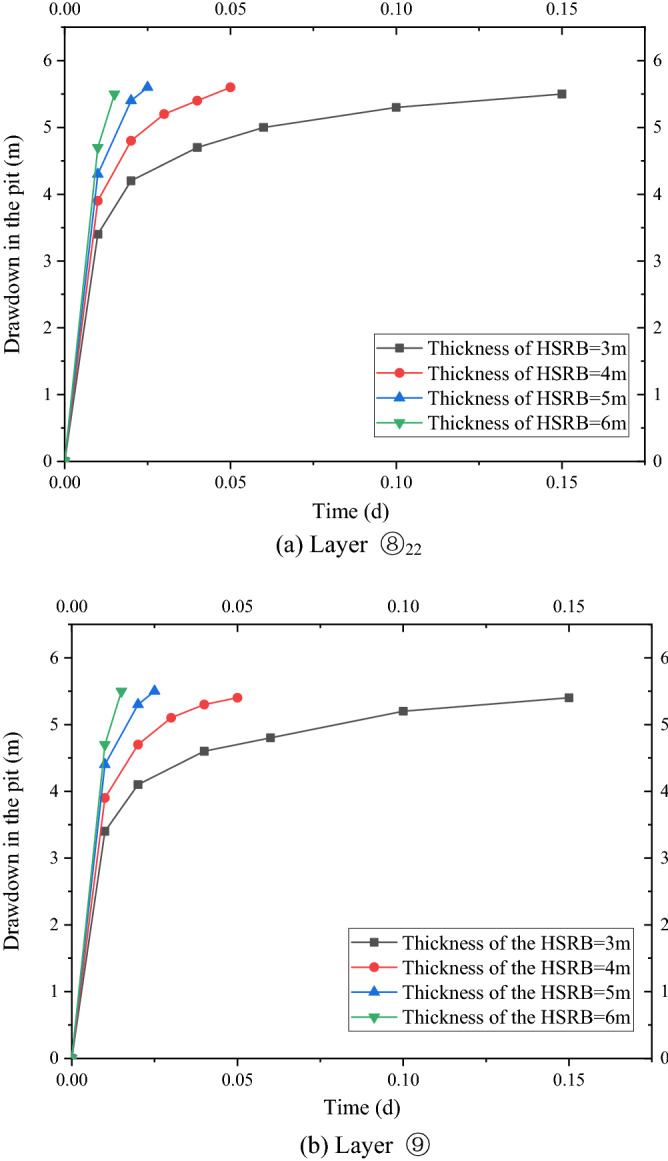
Drawdown–time curve in the pit with different HSRB thicknesses.

The drawdown time curves of the water level in the pit under four working conditions with different thicknesses of the HSRB based on the numerical simulation results are shown in Figs. 11a and 11b. Evidently, the variation law of the drawdown time curve in the two soil layers was consistent. Minimal time was needed to achieve the design drawdown with the increase of the HSRB thickness. Before the design drawdown was reached, the drawdown of the water level in the foundation pit was greater when the HSRB was thicker. When water level in the pit reached the designed drawdown in layer ⑧_22_, drawdown in the pit in layer ⑨ can reach at least 5.4 m, which can meet the design drawdown of the anti-gushing calculation in layer ⑨. Therefore, the pumping time only needed to meet the drawdown requirements of the water level in layer ⑧_22_. In case 8, the pumping well should work for approximately 0.15 d when HSRB thickness was 3 m and the drawdown in the pit met the design requirements. In case 9, the pumping well should work approximately 72 min when HSRB thickness was 4 m and the drawdown in the pit met the design requirements. The pumping well must work for approximately 36 min when the horizontal curtain thickness was 5 m in working condition 10 and drawdown in the pit met the design requirements. The pumping well should work for approximately 22 min when HSRB thickness of working condition 11 was 6 m and drawdown in the pit met the design requirements. Pumping time for the foundation pit dewatering to reach the design water level was reduced by approximately 50% when the thickness was increased by 1 m when HSRB thickness was 3 m to 6 m. Accordingly, the pumping efficiency approximately doubled.

The working efficiency of the pumping well was higher when the HSRB was thicker. However, the improvement range of the working efficiency of the pumping well decreased with the increase of thickness. Therefore, designing a 3D curtain in an actual project entails comprehensive consideration of the effect and cost should be comprehensively considered to select the most appropriate horizontal thickness.

**Figure 12:**
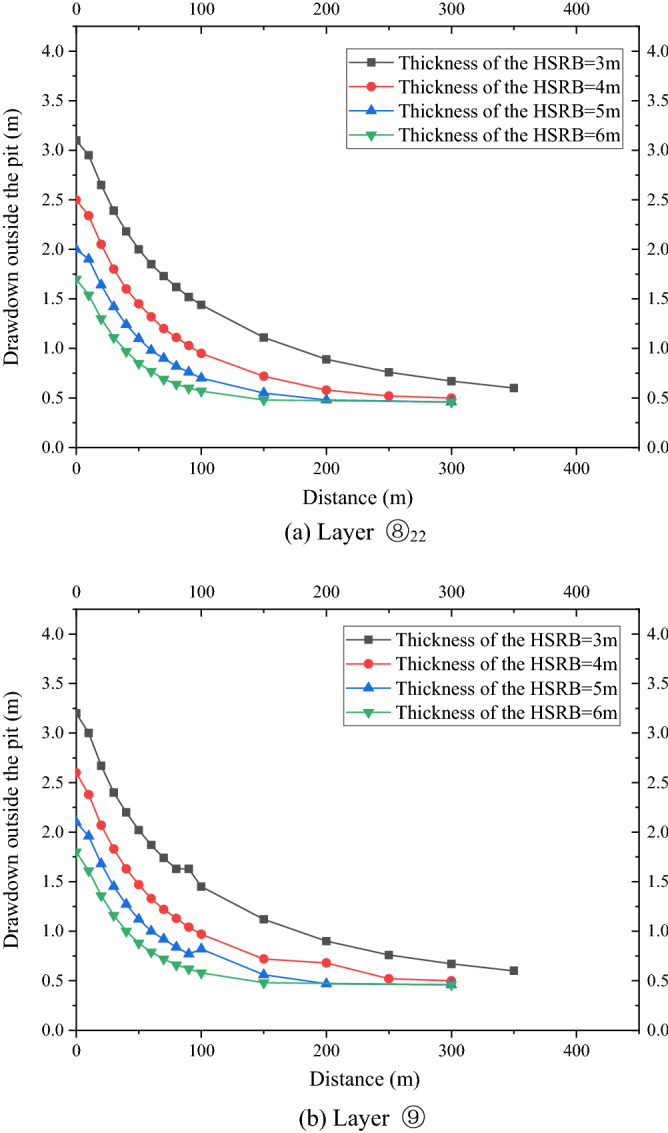
Drawdown–distance curve outside the pit with different HSRB thicknesses.

The drawdown–distance curves of the four working conditions with different HSRB thicknesses based on the numerical simulation results are shown in Fig. 12a and b. The drawdown value and trend of the drawdown rate of water level outside the pit were the same with layers ⑧_22_ and ⑨. Evidently, this study only analyzed the drawdown variation of the water level outside the pit with the distance in layer ⑧_22_. The water level decreased deeper at the same distance outside the pit when the HSRB was thicker. The change trend of the drawdown–distance curve was consistent under the four conditions, and the slope of the curve was the same. That is, the drawdown rate with distance was the same. The maximum drawdown outside the pit was 3.1 m when HSRB thickness was 3 m, and it reached a stable drawdown of 0.6 m at 350 m away from the pit. The maximum drawdown outside the pit was 2.5 m when HSRB thickness was 4 m, and it reached a stable drawdown of 0.52 m at 250 m away from the pit. The maximum drawdown outside the pit was 2.0 m when HSRB thickness was 5 m, and it reached a stable drawdown of 0.53 m at 200 m away from the pit. The maximum drawdown outside the pit was 1.7 m when HSRB thickness was 6 m, and the stable drawdown was 0.51 m at 200 m away from the pit. The lifting effect was significant when HSRB thickness increases from 3 to 4 m. Thereafter, the curves began to be closer with the increase in thickness, and a trend of gradual coincidence was observed. That is, increasing the horizontal curtain thickness will no longer significantly improve the foundation pit dewatering effect when the horizontal curtain thickness relatively increased.

Therefore, the time required for the drawdown in the pit to reach the design value, the maximum drawdown outside the pit, and the influence range of drawdown outside the pit were reduced with an increase in HSRB thickness. The effect of increasing curtain thickness was considerably evident when HSRB thickness was small. The effect of increasing curtain thickness was minimally evident when thickness increased to a certain extent. Therefore, the effect and cost should be comprehensively considered in the engineering design of 3D curtain, and the most appropriate horizontal curtain thickness must be selected.

### Influence of HSRB hydraulic conductivity on dewatering

The analysis results of the influence of the position and thickness of the horizontal seepage reducing curtain on the dewatering effect of the deep foundation pit of the 3D curtain-well group system and the existing 86 m-deep vertical curtain indicate that adding an HSRB with the roof buried depth of 82 m and thickness of 6 m had the best effect on the control of dewatering period and the decline in the underground water level outside the pit in the dewatering process of the deep foundation pit.

Working conditions 13 to 15 and 12 were compared and analyzed to study the influence of hydraulic conductivity of HSRB on deep foundation pit dewatering. Four pumping wells under four working conditions were pumped at a pumping rate of 2500 m^3^/d, the buried depth of HSRB roof was 82 m, and HSRB thickness was 6 m. The form of the 3D curtain formed by horizontal and vertical curtains was inner-wrapped 3D curtain. The hydraulic conductivities of working conditions 13, 12, 14, and 15 were 1 × 10^−2^, 5 × 10^−3^, 1 × 10^−4^, and 5 × 10^−4^ cm/s, respectively, as shown in Table 7.

**Table 7:** Working conditions of HSRB with different hydraulic conductivities.

Working condition	Buried depth of HSRB roof (m)	Thickness of HSRB (m)	Hydraulic conductivity of HSRB (cm/s)	Pumping rates (m^3^/d)
13	82	6	1 × 10^–2^	2500
12	82	6	5 × 10^–3^	2500
14	82	6	1 × 10^–3^	2500
15	82	6	5 × 10^–4^	2500

**Figure 13:**
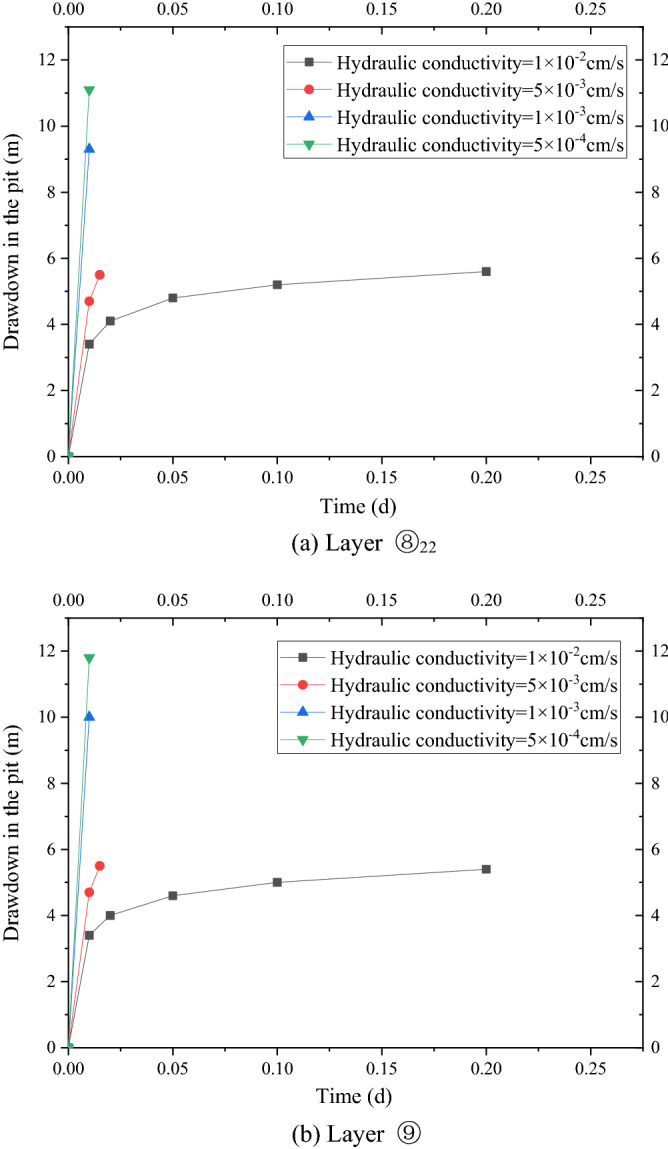
Drawdown–time curve in the pit with different hydraulic conductivities.

**Figure 14:**
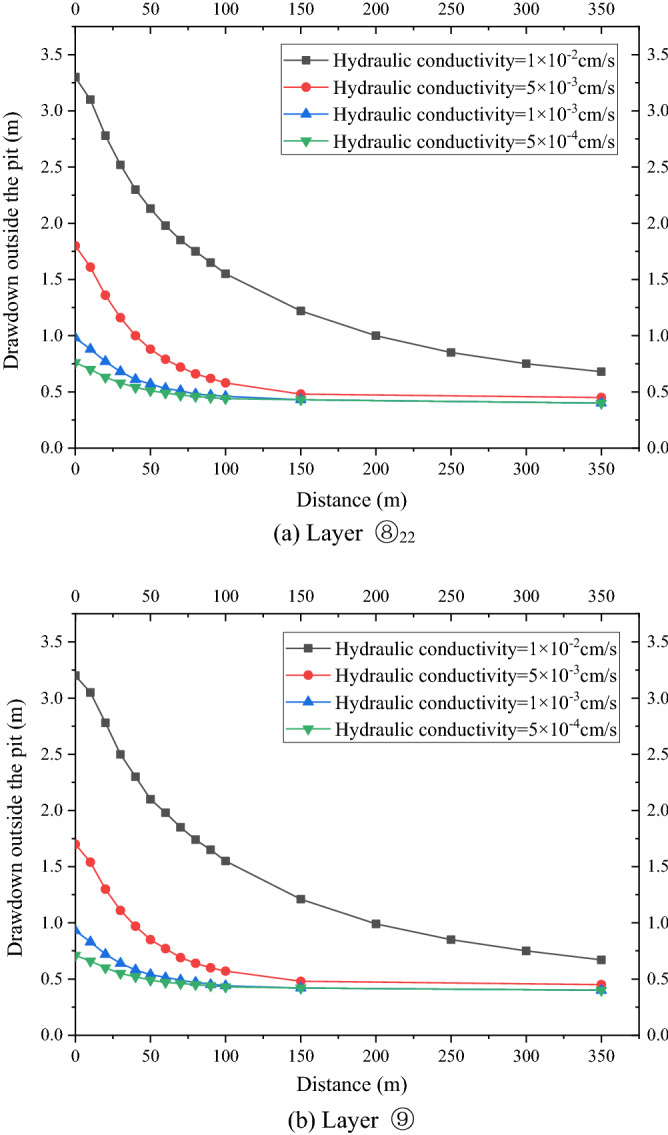
Drawdown–time curve outside the pit with different hydraulic conductivities.

Drawdown–time curves of the water level in the pit under four conditions with different hydraulic conductivities based on the results of the numerical simulation are shown in Fig. 13a,b, and the drawdown-to-distance curves outside the pit are shown in Fig. 14a,b. As shown in Fig. 13a,b, the time required for the water level in the pit to reach the design drawdown decreased with a decrease in the hydraulic conductivity of the HSRB. When water level in the pit reached the designed drawdown of layer ⑧_22_, the drawdown of the water level in the pit of layer ⑨ can reach at least 5.5 m, which met the design drawdown of the surge calculation of layer ⑨. Therefore, the pumping time only needed to meet the seepage reduction demand of layer ⑧_22_. A total of 0.20 d was needed to reach the design drawdown when the hydraulic conductivity of HSRB was 1 × 10^−2^ cm/s. A total of 22 min was needed to reach the design drawdown when the hydraulic conductivity of HSRB was 5 × 10^−3^ cm/s. The water level in the foundation pit decreased rapidly when the hydraulic conductivity of HSRB was 1 × 10^−3^ cm/s, and it reached 9.3 m in 15 min. The drawdown of water level in the foundation pit was faster than that in working condition 14 when the hydraulic conductivity of HSRB was 5 × 10^−4^ cm/s, and it reached 11.1 m in 15 min. Only 5% of the hydraulic conductivity of 1 × 10^−2^ cm/s was needed to reach deeper drawdown when the hydraulic conductivity of HSRB was below 1 × 10^−3^ cm/s. Therefore, dewatering time can be considered a secondary factor, and drawdown outside the pit was mainly considered. That is, the impact of the foundation pit dewatering on the environment outside the pit was considered.

As shown in Fig. 14a,b, the drawdown value and trend outside the pit of the two layers were the same. With a decrease in the hydraulic conductivity of HSRB, the maximum drawdown of the water level outside the pit was smaller, and the influence of foundation pit dewatering on the outside of the pit became smaller. Therefore, only the variation in the drawdown of water level outside the pit of layer ⑧_22_ with the distance was analyzed. When the hydraulic conductivity of HSRB was 1 × 10^−2^ cm/s, the maximum drawdown of the water level outside the pit was 3.2 m, and stable drawdown of the water level was 0.67 m at 350 m away from the pit. When the hydraulic conductivity of HSRB was 5 × 10^−3^ cm/s, the maximum drawdown of the water level outside the pit was 1.7 m, and stable drawdown of the water level was 0.48 m at 150 m away from the pit. When the hydraulic conductivity of HSRB was 1 × 10^−3^ cm/s, the maximum drawdown of the water level outside the pit was 0.93 m, and stable drawdown of the water level was 0.42 m at 150 m away from the pit. When the hydraulic conductivity of HSRB was 5 × 10^−4^ cm/s, the maximum drawdown of the water level outside the pit was 0.71 m, and the stable drawdown of the water level was 0.42 m at 150 m away from the pit. The curves of each working condition were increasingly closer with a decrease in hydraulic conductivity of horizontal curtain, particularly when the hydraulic conductivity of HSRB was below 1 × 10^−3^ cm/s. Accordingly, the curves began to overlap, which indicates that the improvement effect of lowering the hydraulic conductivity when it was below 1 × 10^−3^ cm/s was no longer evident.

Therefore, the time required for water level in the pit to reach the design drawdown was reduced, the maximum drawdown outside the pit was decreased, and the impact of foundation pit were decreased with a decrease in the hydraulic conductivity of HSRB. The improvement effect was evident when the hydraulic conductivity of HSRB was reduced from 1 × 10^−2^ cm/s to 5 × 10^−3^ cm/s. However, the improvement effect of further reduction of hydraulic conductivity on the drawdown and influence range of drawdown outside the pit was no longer evident when hydraulic conductivity of HSRB was reduced below 1 × 10^−3^ cm/s. Therefore, blindly pursuing lower permeability is no longer necessary when using the MICP technology to reduce the permeability of sand.

## Discussions

### Fifth foundation pit seepage modes

**Figure 15:**
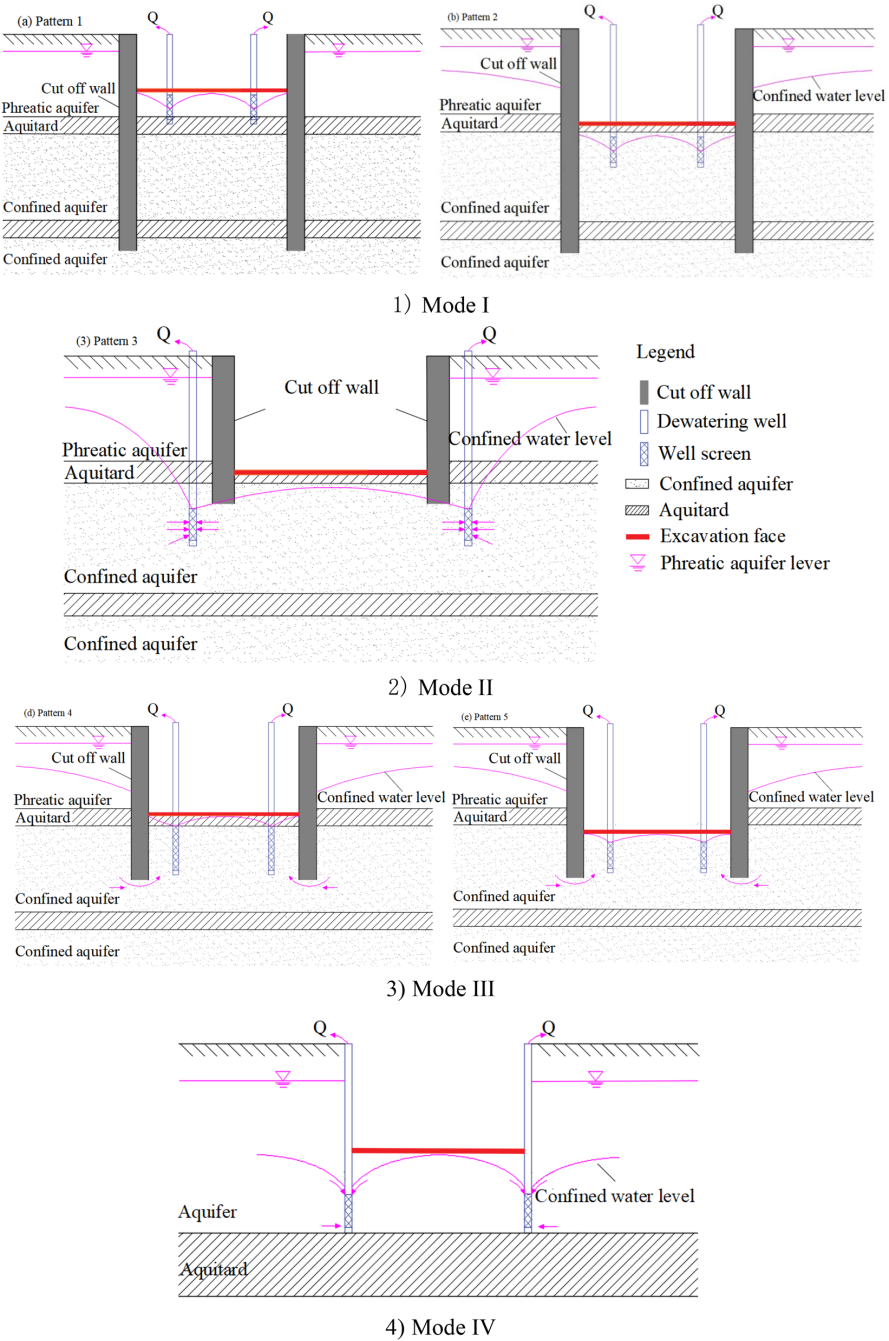
Conceptual model of four summarized seepage modes.

In deep foundation pit dewatering, vertical curtains are often adopted in Multi-Aquifer and Multi-Aquitard (MAMA) to control drawdown inside and outside pit. Wu et al. (2003) summarized three seepage modes of foundation pit considering vertical penetration condition in MAMA. The seepage mode outside curtain during portal and export dewatering for a shield machine in MAMA was defined as the fourth seepage mode. The four seepage modes (Fig. 15) were based on vertical curtain and the penetration conditions of MAMA.

1. Mode I (Curtain penetrating shallow aquifers and partially penetrating bottom aquitard of the target aquifer of a MAMA).
  In the mode, vertical curtain cuts off all the target aquifers (should be dewatered) of MAMA. The bottom of the vertical curtain penetrated all shallow aquifers and partially penetrated the top aquitard of the lowest confined aquifer that should be dewatered. The side and bottom boundaries of the water flow were the cutoff wall and aquitard, respectively. Water level in the pit was lowered using pumping wells within the boundaries. Three sub-modes were defined according to the dewatered aquifers.
  Mode 1–1: The excavation face located in the phreatic aquifer and underlying confined water pressure satisfied anti-gushing conditions, and vacuum well point, waterway, and shallow pumping well were arranged to drain the aquifers.
  Mode 1–2: The excavation face located in the phreatic aquifer and underlying confined water pressure did not satisfy anti-gushing conditions, and pumping wells were arranged to drain the phreatic aquifer and lower the water level of the confined aquifer.
  Mode 1–3: The excavation face located in the confined aquifer, the top aquitard of the shallow confined aquifer was excavated, and pumping wells were arranged to drain the phreatic aquifer, shallow confined aquifers, and exposed confined aquifer.
2. Mode II (Curtain penetrating shallow aquifers and partially penetrating top aquitard of a MAMA).
  The vertical curtain penetrated the top aquitard of the deepest confined aquifer that should be dewatered. Vertical curtain penetrated and cut off all shallow aquifers. However, no vertical curtain penetrated the deepest confined aquifer that needed to lower water level. Three sub-modes were defined according to the dewatered aquifer. Modes 2–1, 2–2, and 2–3 were the same as modes 1–1, 1–2, and 1–3 for shallow aquifers. Separated pumping wells had to be arranged in the deepest confined aquifer inside or outside the pit to lower the water level. Seepage mode included the water flow in shallow aquifers cut off by walls, underlying water flow in deep aquifer without cutoff walls, and cross-flow between the shallow and deep aquifers.
3. Mode III (Curtain penetrating shallow aquifers and partially penetrating deep target aquifers of a MAMA).
  The cut off wall penetrated the shallow MAMA aquifers and partially penetrated deep confined aquifer that should be dewatered. Cut off shallow aquifers can be divided into three sub-modes similar to those defined in Modes I and II. According to the depth of the cut off wall and pumping well filter tubes bottom, three pumping well arrangement patterns were formed for the underlying partially penetrated curtain, namely, the (1) entire filter tube enveloped by curtain pattern, (2) filter tube partially enveloped by curtain and part of the filter tube exposed a curtain pattern, and (3) all filter tube exposed curtain pattern. Nine seepage modes were combined: Mode III-1i(i = 1,2,3), Mode III-2j(j = 1,2,3), and Mode III-3 k (k = 1,2,3) in curtain pattern. Water flow occurred in cut off shallow aquifers and partially cut off deep aquifers, as well as the leakage and crossflow between the shallow and deep aquifers.
4. Mode IV (Pumping outside curtain and nearby shield tunnel type).
  When a shield machine entered or left the portal or expose working pit, dewatering was occasionally necessary to control water pressure and leakage of reinforced soil. Pumping wells were arranged outside the cut off wall and near the shield machine and tunnel. The cutoff wall and tunnel influenced the water flow as boundaries. The dewatering type was defined as Mode IV.

**Figure 16:**
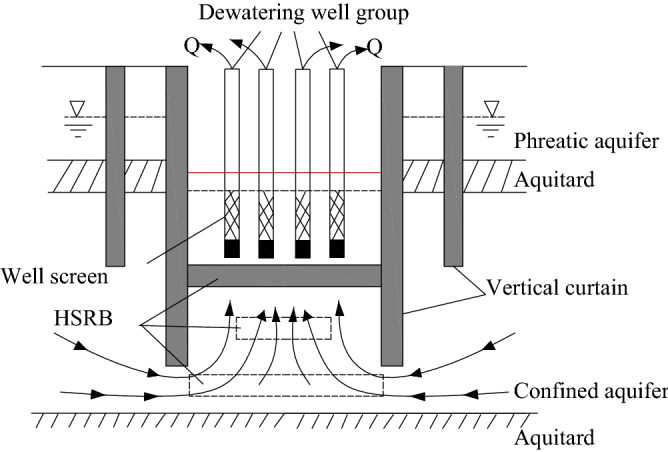
Conceptual model of three-dimensional curtain-well group system.

The current four seepage modes were widely used. However, vertical curtain may fail to effectively cut off all confined aquifers of MAMA and achieve the designed dewatering effects when the confined aquifer was considerably thick or buried substantially deep. The current study introduced an HSRB as man-made aquiclude to decrease hydraulic conductivity in deep confined aquifer. The man-made HSRB belongs to an anti-seepage body formed by various construction technologies at a certain depth of confined aquifers. Horizontal curtain was previously used as a complete impermeable curtain, the seepage of which belongs to Mode I. Horizontal curtain required advanced construction technology and was costly. The man-made HSRB was formed to improve the vertical water blocking effect rather than a water proof curtain. HSRB reduced the hydraulic conductivity of aquifer soil by grouting and other techniques to weaken the hydraulic connection inside and outside the pit. The conceptual model is shown in Fig. 16.

HSRB decreased the hydraulic conductivity of the soil by grouting in confined aquifer, formed an HSRB with certain thickness to reduce seepage in foundation pit dewatering, increased hydraulic gradient in the curtain body, reduced outlet water pressure and effectively controlled seepage flow. This method was economical, simple, and easy to realize compared with the complete impervious curtain. Seepage occurred inside the body and bore substantial hydrodynamic force to decrease the outlet water pressure.

According to the relative position of HSRB and vertical curtain, the combination can be divided into two categories: separated and non-separated. Non-separated can be subdivided into three types: inner-wrapped, transitional, and flush.

1. Mode V-1 (Separated 3D curtain): HSRB was separated from the suspension waterproof curtain, as shown in Fig. 17a. HSRB was equivalent to setting a certain area as man-made aquiclude in the aquifer below the vertical curtain at certain depths. This mode was formed in the cases that the HSRB was deeper than vertical curtain and formed after the vertical one.

**Figure 17:**
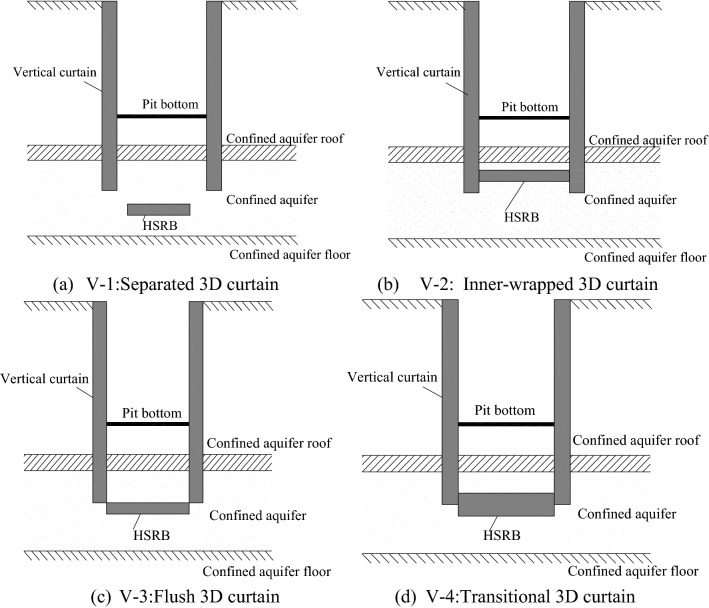
Combination sub-mode of vertical curtain and HSRB.
2. Mode V-2 (Inner-wrapped 3D curtain): The HSRB was inside the vertical curtain, as shown in Fig. 17b, similar to a box that only allowed a small amount of water to seep at the bottom. Given that HSRB was completely within the vertical curtain range, vertical curtain can prevent grout from spreading into surrounding groundwater when grouting technology was used to form HSRB. Thus, environmental pollution would be reduced.
3. Mode V-3 (Flush 3D curtain): The top of the HSRB connected with the vertical curtain, as shown in Fig. 17c. This 3D curtain was approximately the same as the inner-wrapped 3D curtain, and both form a partially closed box with the vertical curtain. The construction of this type of scheme can avoid the influence of the vertical curtain and can be realized through some processes outside the pit. However, the influence of the groundwater seepage field outside the pit should be considered.
4. Mode V-4 (Transitional 3D curtain): Given that the horizontal body had a certain thickness, the top of the horizontal body was within the depth of the vertical curtain and the bottom plate was outside the range of the vertical curtain depth. This 3D curtain that transitioned from an inner envelope to a level was defined as a transition 3D curtain, as shown in Fig. 17d.

**Figure 18:**
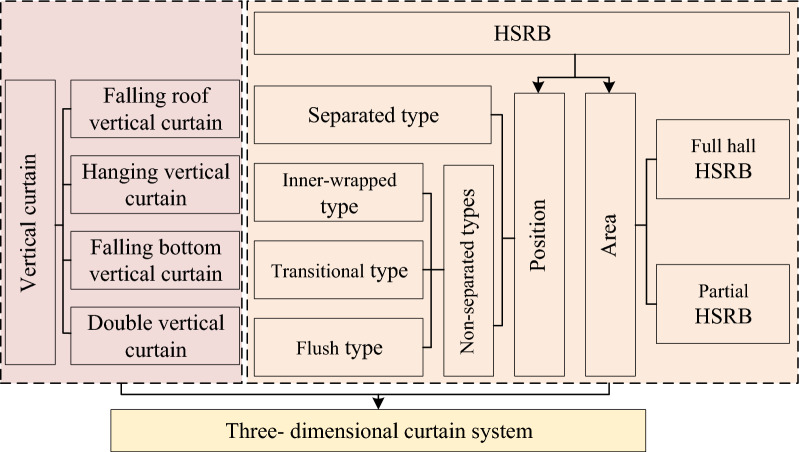
Framework of the 3D curtain concept system.

Suspended vertical curtain and HSRB were combined in foundation pit dewatering, as shown in Fig. 18. The HSRB was divided into full and local HSRB. Full HSRB contacted with vertical curtain to form a partially closed box. The partially HSRB did not contact vertical curtain to form a non-closed solid curtain.

When the survey data showed numerous underlying partial impermeable zones or weakly permeable water bodies in the confined aquifer, these natural horizontal water-tight structures can be utilized to control the design cost and construction difficulty (i.e., local horizontal curtains were set around the weak water-permeable body).

### HSRB construction method

The man-made HSRB can be considered a type of permeable horizontal curtain. At present, the main forms of vertical curtain include diaphragm wall, Soil Mixing Wall (SMW) method, cement–soil mixing method, and high-pressure jet grouting method. Diaphragm wall can be used as retaining structure, and it was widely used as vertical curtain in deep foundation pit. However, its cost was high when used as curtain. The SMW method can also combine the functions of waterproof and retaining structure by mixing cement slurry with the original soil and inserting an H-shaped steel. The SMW method had short construction period, low environmental impact, good seepage insulation effect, and relatively low cost. This method included dry and wet construction processes with short construction period and low requirements for construction conditions. However, the working depth of the method was limited. The high-pressure jet grouting method can combine support row piles or soil nail walls to achieve waterproof and retain functions. It cut the soil mass through the cement slurry ejected from the nozzle, the undisturbed soil and slurry were mixed to form cement soil, and hydraulic conductivity of cement–soil was considerably lower than that of the undisturbed soil mass. This method was easy to construct and construction equipment required simple. However, guaranteeing construction quality was difficult when the construction depth was considerably deep. Freezing method had immense advantages in the construction of complex and special strata, including convenient construction and recoverable engineering equipment. The freezing method was better when the excavation was deeper. However, water cannot be pumped during the freezing period. Special attention should be given to the overall stability, freezing, and thermal insulation of the curtain.

The HSRB construction method should be selected according to engineering, geological, and economic conditions. The HSRB was permeable instead of impermeable curtain, and the main purpose was to reduce the hydraulic conductivity of the target aquifer from one to two orders of magnitude. Grouting method was often suggested. However, the high pressure of jet cement destroyed the structure of aquifer and aquitard, and partial leakage cannot be avoided. The connection of the different piles was difficult when depth was large. This study suggested the MICP grouting technology to form HSRB with grout of *Bacillus pasteurella* and cementing fluid (CaCl_2_ solution, urea solution), which has minimal impact on the environment. The structure of the aquitard and aquifer was not destroyed.

The traditional materials used in the traditional horizontal curtain forming method, such as cement and lime cementitious materials, caused adverse effects on the ecological environment of groundwater. Moreover, traditional grouting materials had difficulty entering the sand layer with small pores, such as layer ⑨ of fine sand in Shanghai. The original structure of the target aquifer was destroyed in traditional technology to form a horizontal curtain. Therefore, using the MICP grouting technology to form environment-friendly horizontal curtain can solve numerous limitations of the traditional horizontal curtain forming method. The formation method of MICP HSRB was presented in another manuscript^*^.

## Conclusions

1. HSRB was added to form a horizontal man-made aquiclude on the bases of vertical waterproof curtain applied in foundation pit dewatering engineering. The vertical curtain, HSRB and pumping wells were designed to work together. The combination included separated and non-separated types.
2. The inner-wrapped, flush, and transitional vertical curtains were equivalent for non-separated HSRB. Non-separated HSRB was better than the separated HSRB. The dewatering effect for separated HSRB was better when the vertical curtain bottom was closer.
3. The time to reach the designed drawdown, the maximum outside drawdown, and the influence range decreased with the increase in HSRB thickness.
4. The effect of increasing the curtain thickness was more obvious when the HSRB was thinner. The effect of increasing the curtain thickness continuously was not very obvious when the HSRB thickness was increased to a certain extent. Therefore, the effect and cost should be comprehensively considered, and the most appropriate HSRB thickness must be selected.
5. The fifth seepage mode was suggested for foundation pit dewatering based on the combination of vertical curtain and HSRB, and it can be referred by similar projects.
